# Understanding the Oxidation Electrochemistry of Adsorbed Eugenol on a Glassy Carbon Electrode Modified with Electrochemically Partially Reduced Graphene Oxide: A Theoretical and Experimental Approach

**DOI:** 10.3390/ijms27052461

**Published:** 2026-03-07

**Authors:** Gastón Darío Pierini, Edgardo Maximiliano Gavilán-Arriazu, Sergio Antonio Rodriguez, Sebastián Noel Robledo, Héctor Fernández, Adrian Marcelo Granero

**Affiliations:** 1 Grupo de Electroanalítica (GEANA), Departamento de Química, Facultad de Ciencias Exactas, Físico-Químicas y Naturales, Instituto para el Desarrollo Agroindustrial y de la Salud (IDAS), Universidad Nacional de Río Cuarto, Río Cuarto 5800, Argentina; 2 Instituto de Bionanotecnología del NOA (INBIONATEC), Universidad Nacional de Santiago del Estero (UNSE), Santiago del Estero 4200, Argentina; maxigavilan@gmail.com; 3 Instituto de Ciencias Químicas, Facultad de Agronomía y Agroindustrias (FAyA), Universidad Nacional de Santiago del Estero (UNSE), CONICET, Av. Belgrano Sur 1912, Santiago del Estero 4200, Argentina; 4 Departamento de Tecnología Química, Facultad de Ingeniería, Instituto para el Desarrollo Agroindustrial y de la Salud (IDAS), Universidad Nacional de Río Cuarto, Río Cuarto 5800, Argentina

**Keywords:** eugenol, nanostructured electrodes, cyclic voltammetry, oxidation mechanism, DFT

## Abstract

The electro-oxidation of eugenol (EUG) natural antioxidant was studied by cyclic voltammetry in phosphate buffer solutions (PBS) of different pH at electrochemically partially reduced graphene oxide (GCE/ePRGO). The voltammetric responses were mainly controlled by adsorption at this modified electrode. Current values were higher at pH 2.0 PBS, therefore, this pH was chosen to perform all experiments. DFT calculations of p*K*a’s and standard potentials defined the possible pathways of eugenol and its oxidation products. These pathways were evaluated through the comparison of voltammetric simulations of adsorbed species with experiments at pH 2.0, which also allowed for the estimation of the values of the kinetic parameters involved in electrochemistry. Our findings suggest a multi-step redox process in which Eugenol is first oxidized to the radical species and then to a cationic product. At this stage, the pathways branch into to methylenquinone and a 4-allyl-1,2-diquinone molecules. 4-allyl-1,2-diquinone is finally reduced in single or double reversible electrochemical step to the hydroquinone species. The present physicochemical work allows for a deeper understanding of the eugenol oxidation mechanism, which was only partially proposed in previous studies.

## 1. Introduction

**Figure 1:**
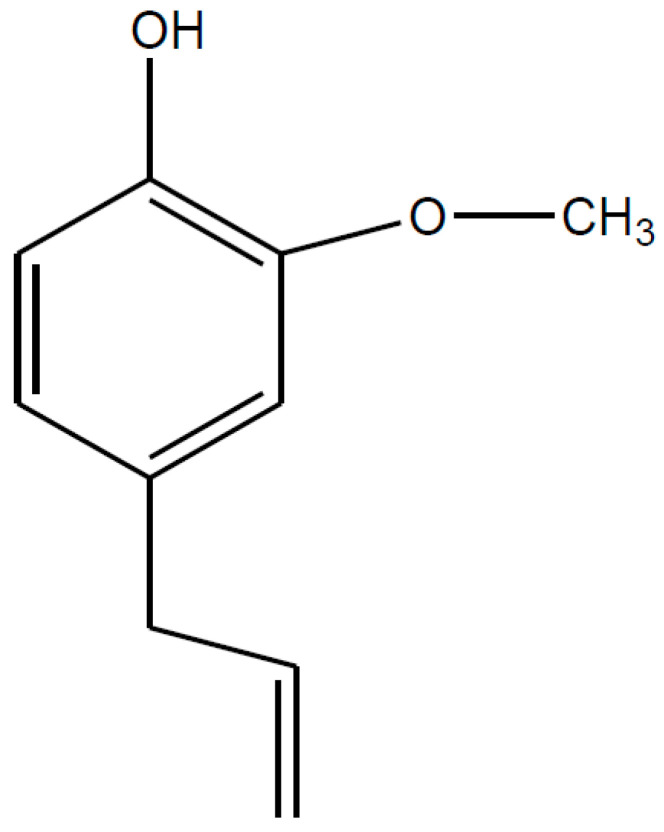
Chemical structure of eugenol.

Eugenol or 4-allyl-2-methoxyphenol (EUG, Figure 1) is a lipophilic antioxidant. It is a liquid at room temperature, pale yellow and oily consistency. It is found mainly in nutmeg, bay leaf, and clove oil [1]. EUG is usually extracted from *Ocimum sanctum* Linn (Tulsi) and *Ocimum basilicum* (Ban Tulsi). The percentage of EUG in the essential oil of *Ocimum sanctum* Linn varies between 40 and 71% [2]. EUG has also antimutagenic, antigenotoxic, antimicrobial, antiviral, antioxidant, and anti-inflammatory properties [3,4,5,6]. EUG has been used since the nineteenth century in the fields of dentistry [7], medicine [8], chemical industry, and food [9], as an antiseptic, anesthetic, stabilizer, and antioxidant. Another interesting use of EUG is as a component of a membrane at an electrode for sensing oxygen in the brain [10]. Also, the use of EUG as a repellent and insecticide is known [11,12].

The EUG electrochemical studies have generally been directed for analytical purposes to the development of methods for its determination in different samples. The studies were conducted on different electrodes, such as at a carbon paste electrode chemically modified with gold nanoparticles (AuNPs) [13], a glassy carbon electrode (GCE) modified with copper-doped AuNPs [14], GCE modified with AuNPs assembled on the surface of poly (diallyldimethylammonium chloride) functionalized graphene-MoS_2_ [15], pencil graphite electrode [16], GCE modified with electrochemically reduced graphene oxide [17], carbon paste electrode (CPE) modified with graphene oxide decorated tin oxide nanoparticles [18], GCE modified with poly-γ-aminobutyric acid [19], chitosan-reduced graphene oxide/polyoxometalates/poly-l-lysine (CS-rGO/P2Mo17Cu/PLL) modified GCE [20], bare carbon nanotube electrode (BCNTE) with sodium dodecyl sulfate (SDS/MWCNT) [21].

On the other hand, in recent years it has increased the use of reduced graphene oxide (RGO)/electrochemically reduced graphene oxide (ERGO) to perform electrochemical studies. RGO is usually obtained by chemical oxidation/exfoliation of graphite and subsequent reduction of GO [22]. RGO is similar to graphene in terms of its electrical, conductivity, thermal, mechanical strength, and flexibility properties [23,24]. These important properties of RGO have led it to be intensively used in electrochemical research [25].

In a previous work, Yildiz et al. [26] proposed an electrochemical sensor based on graphene modified CPE, and the mechanism of its electro-oxidation was unveiled using theoretical calculations and mass spectrometric analysis at graphene CPE. EUG was adsorbed to the electrode surface. The authors proposed two main oxidation products that coexist in the electrode surface. Nevertheless, this study is unable to provide a comprehensive explanation for the nature of the electrochemical phenomena observed in the consecutive cyclic voltammograms. On the other hand, Wang et al. [17] studied the electrochemistry of EUG at GCE/ePRGO. The authors described an adsorbed couple in consecutive cyclic voltammograms corresponding to the reversible redox transition between 4-allyl-1,2-quinone and 4-allyl-1,2-benzenediol. However, no theoretical study is done to demonstrate the nature of that redox pair.

On the other hand, to date, there are only a small number of reports on the use of electrochemical eugenol sensors that are based on electrodes modified with graphene (and its derivatives). The electrochemical behavior of the main oxidation peak remains to be elucidated, with some authors proposing diffusion-controlled electrode processes [17,18] and others adsorption-controlled processes [15,26].

Despite the extensive research conducted on the electrooxidation mechanism of eugenol, the nature of the peaks observed in consecutive cyclic voltammetry experiments remains to be fully elucidated. This paper describes first, the origin of the electro-oxidation of the EUG anodic oxidation product at GCE’s modified with electrochemically partially reduced graphene oxide (GCE/ePRGO). Second, the electrochemistry response in 0.2 M phosphate buffer solutions (PBS) of different pH’s. The EUG oxidation product (EUGOP) electro-oxidation at GCE/ePRGO was mainly controlled by adsorption. Current values were higher at pH 2.0 PBS than at other pH’s. Therefore, this pH was chosen to perform the subsequent studies. The EUGOP gives place to a quasi-reversible redox couple formation, which is assigned to the 4-allyl-1,2-diquinone/4-allyl-1,2-dihydroquinone redox couple. From cyclic voltammograms recorded in pH 2.0 PBS at different scan rates, the kinetics parameters of the EUGOP such as the overall heterogeneous rate constant (*k_s_*) and the anodic transfer coefficient (1 − α) were determined. Finally, theoretical studies were conducted to unveil the oxidation mechanism of EUG. This is why with the support of experimental measurement and simulations, the present work aims for a deeper understanding of the eugenol oxidation mechanism in an aqueous solution, which was partially proposed in previous studies.

## 2. Results and Discussion

### 2.1. Electrode Characterization

The surface morphologies of the GO film before and after its electrochemical reduction have been characterized by SEM and EIS. As mentioned in the previous section, the GO reduction was performed using CV. Figure S1a in Supplementary Material (SM) displays the reductive consecutive cycles of GO. Upon examination of the CV profile, a distinct and prominent cathodic peak was observed in the initial potential cycle at about −1.5 V, with the absence of anodic peaks following the reversal of the potential scanning direction. This phenomenon substantiates the irreversible nature of the verified reduction process. The aforementioned cathodic peak has been ascribed to the reduction of oxygenated functional groups present throughout the graphene sheets, including epoxy, peroxy, and aldehyde groups [27].

The surface morphology of the different stages of electrode surface construction was investigated using SEM. Figure S1 displays typical SEM images for (B) GCE and (C) GCE/ePRGO at different magnifications. As expected, significant changes in surface roughness are evident when comparing GCE and GCE/ePRGO. The SEM images obtained for the modified electrodes exhibit similarities with those obtained by other researchers in the field [28,29].

On the other hand, Figure S2 shows the experimental Nyquist plots obtained for the bare GCE (black dotted), the GCE/GO (red dotted), and the GCE/ePRGO (blue dotted). The impedance plots were fitted using an equivalent electrical circuit comprising the following components: R_1_, representing the solution resistance between the working and reference electrodes; R_2_, the charge transfer resistance; and W_S_, the Warburg element, which represents the linear diffusion of the redox couple to the electrode surface as shows Figure S2. Q_1_ and Q_2_ correspond to a constant phase element. Values of R_2_ of 196, 520, and 50 Ω were obtained for the bare GCE, the GCE/GO, and GCE/ePRGO, respectively. This means that there is less resistance to charge transfer at the GCE/ePRGO electrode surface.

### 2.2. Electrochemical Behavior of EUG at GCE, GCE/GO, and GCE/ePRGO

**Figure 2:**
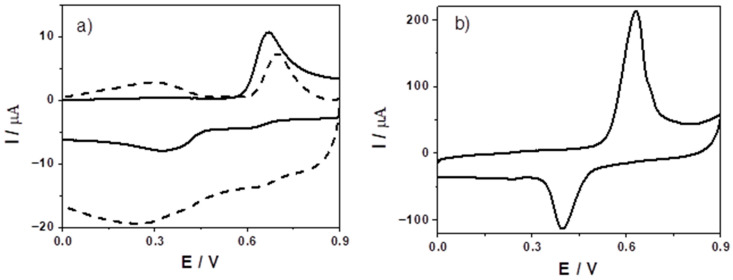
Cyclic voltammograms of 5 × 10^−4^ M EUG in 0.2 M pH 2.0 PBS at (**a**) GCE (solid line) and GCE/GO (dashed line), and (**b**) at GCE/ePRGO. v = 0.100 V s^−1^.

The electrochemical behavior of EUG was studied using the three different types of carbon-based electrodes, to show the main characteristic current response in each of them. Figure 2 shows the cyclic voltammograms of EUG at (a) bare GCE (solid line) and GCE/GO (dashed line) and (b) GCE/ePRGO in pH 2.0 PBS. EUG shows an anodic peak centered at a potential (E_p,a_) of 0.67 V at bare GCE (Figure 2a). In addition, a reduction peak is clearly defined at a potential of about 0.34 V when the direction of the potential sweep was reversed. Both anodic and cathodic peaks show a characteristic profile of a redox process mainly controlled by diffusion. The E_p,a_ of EUG increased slightly at GCE/GO compared with bare GCE (about 0.70 V), while the anodic peak current (I_p,a_) decreased by about 30%. This fact may be explained by considering that GO has oxygen groups (hydroxyl, epoxy, and carboxylic groups) in its structure that produce a significant separation between the sheets that compose it causing this compound to be a poor electrical conductor [30]. When GCE/ePRGO is produced by the electrochemical reduction of GCE/GO (Figure S1a), it was observed that not only the EUG E_p,a_ decreases (about 0.04 V) but also a huge increase in I_p,a_ was obtained, approximately 20 times higher than that obtained at the bare GCE. This is in consonance with EIS results presented previously, where charge transfer resistance on GCE/ePRGO surface is the smallest of the three electrodes. Moreover, the reaction in GCE/ePRGO shows a characteristic adsorptive behavior, this is verified plotting the anodic peak current vs. the sweep rate, where a linear behavior is observed (Figure S3). Previous to these results we examined the adsorption times of EUG in GCE/ePRGO at Open-Circuit Potential conditions, and it was found that 30 min is enough to promote the maximum adsorption for a given concentration of EUG in bulk solution (Figure S4) with a saturation concentration of 5 × 10^−3^ M.

To study in detail the oxidation mechanism of EUG we choose working with GCE/ePRGO. This was not an arbitrary selection since when the process is dominated by adsorption, voltammetry gives a much more direct reading of the intrinsic electrochemistry of the compound. For example, if the species is adsorbed onto the surface, the amount of material participating in the reaction is finite and known; also, it does not depend on concentration gradients within the volume, there is no transport from the bulk (or is negligible) and the signal depends almost exclusively on electron kinetics, the formal potential and surface interactions.

### 2.3. Effect of pH on the EUG Electrochemical Oxidation at GCE/ePRGO

**Figure 3:**
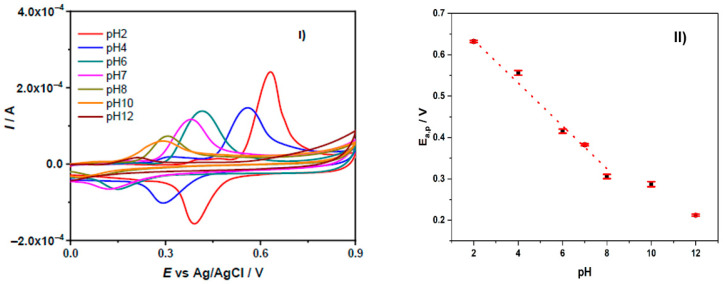
(**I**) Cyclic voltammograms of 5 × 10^−4^ M EUG in PBS at GCE/eRPGO at different pH values. (**II**) Dependence of the anodic peak potential with the pH. v = 0.100 V s^−1^.

To find the best EUG signal conditions and search for first mechanistic clues, a voltammetric study was conducted at different pHs. Cyclic voltammograms of EUG at GCE/ePRGO show a main oxidation peak in the potential region from 0 to 0.9 V at different pH. The pH was varied between 2.0 and 12.0 (Figure 3I). The main oxidation peak is shifted at less anodic potentials as the pH increases. The variation of E_p,a_ with pH was ∂E_p,a_/∂pH = −(0.054 ± 0.002) V/decade (linear correlation coefficient, r = 0.9950), indicating that the same number of electrons and protons are involved in the electrode process (Figure 3II). In addition, this plot shows a breakpoint at pH 10, in very good agreement with the value of EUG apparent acidity constant, K_a_^app^, previously determined by us from UV-Visible spectroscopic measurements, pK_a_^app^ = 10.20 [31]. Moreover, from Figure 3I,II can be inferred that the highest currents for the EUG electro-oxidation were obtained at pH 2.0. This behavior can be explained by considering that protons are involved in the electrode process, making the protons less available as the pH increases. Since we do not want that proton concentration being a limitation and also since a higher current implies greater sensitivity of electrochemical processes, we have chosen pH 2 as the pH of study.

### 2.4. Consecutive Cyclic Voltammograms of EUG at GCE/ePRGO in pH 2.0 PBS

**Figure 4:**
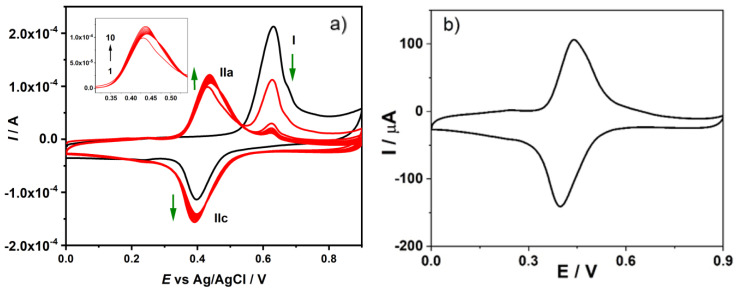
(**a**) Successive cyclic voltammograms of 5 × 10^−4^ M EUG in pH 2.0 PBS at GCE/ePRGO; (**b**) Cyclic voltammogram recorded in pH 2.0 PBS (blank solution) after successive cycles as described in a). v = 0.100 V s^−1^. t_acc_ = 1800 s; E_acc_ = OCP ≈ 0.21 V.

**Scheme 1:**
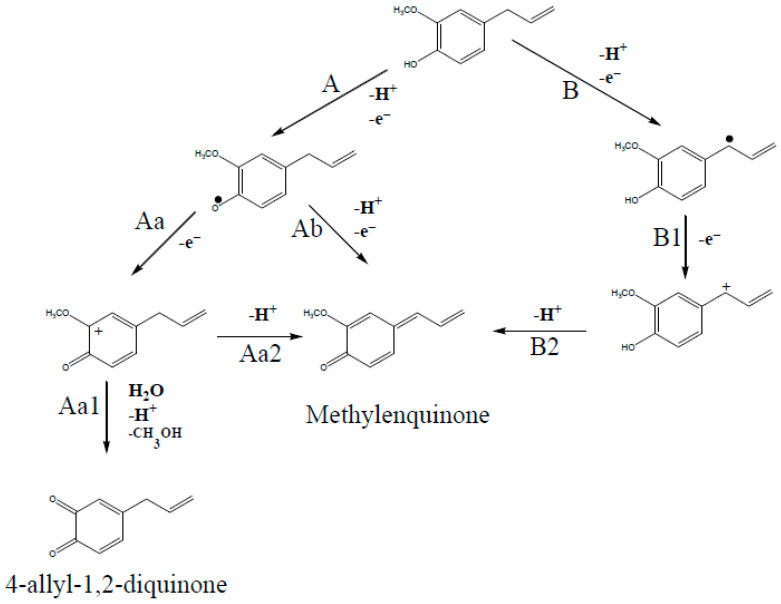
Oxidation mechanism of peak Ia for EUG at GCE/ePRGO in pH 2.0 PBS.

Performing successive voltammetric cycles is a way to trace the electrochemical reactions of EUG oxidation products. Figure 4a shows ten successive cyclic voltammograms recorded for EUG at GCE/ePRGO in pH 2.0 PBS. In successive sweeps, the main oxidation peak (peak Ia) decreases and a new system of quasi-reversible peaks appears in a region of potentials smaller than those of the main oxidation peak (peaks IIa/IIc). Peaks IIa/IIc increase as the peak Ia decreases. This behavior demonstrates that processes involved at the main oxidation peak include homogeneous chemical reaction/s coupled to the initial charge transfer. Thus, a small peak remains in the region of the main oxidation peak and the peaks IIa/IIc show their maximum current values. After performing these experiments, the electrode was rinsed with the blank solution and transferred to another electrochemical cell containing only the supporting electrolyte (blank solution). Under these conditions, the IIa/IIc system shows peak currents slightly lower than those found in the presence of EUG (Figure 4b). Peak Ia of EUG in pH 2.0 PBS can be explained by considering a general reaction mechanism previously reported (Scheme 1) [14,16,26].

In the first stage of EUG oxidation, according to this mechanism, the peak Ia corresponds to the loss of an electron and a proton (from the phenol group), generating a phenoxy radical (route A). Then, the phenoxy radical loses an electron generating the corresponding carbocation (route Aa). Finally, the carbocation loses a proton and a methoxy group and through the incorporation of a water molecule generates the 4-allyl-1,2-diquinone (route Aa1). The carbocation can lose a proton to give the methylenquinone (route Aa2). Another possible alternative route is for the phenoxy radical to lose a proton and an electron to give also methylenquinone (route Ab).

On the other hand, the main oxidation step of EUG can occur due to the loss of an electron and a proton resulting in the formation of an allylic radical (route B). Then, this radical also gives rise to the formation of methylenquinone through the loss of an electron (generating a carbocation) and a proton (routes B1 and B2, respectively). In the computational section we will contrast this mechanism with DFT results.

**Scheme 2:**
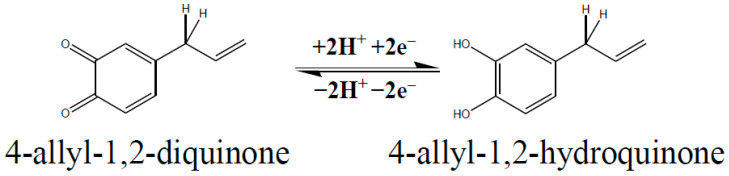
Quasi-reversible redox couple of peaks IIa/IIc for EUG electro-oxidation at GCE/ePRGO in pH 2.0 PBS after performing successive cyclic voltammograms. In the presence of EUG.

Both 4-allyl-1,2-diquinone and methylenquinone could be formed in the electrochemical oxidation of EUG. However, the 4-allyl-1,2-diquinone would be responsible for the appearance of peaks IIa/IIc during the successive potential sweeps. It is well known that the catechol/*o*-diquinone redox couple in an acidic medium shows a typical quasi-reversible behavior [14,16]. Therefore, the system of peaks IIa/IIc may correspond to the redox couple shown in Scheme 2.

Thus, when the direction of the potential sweep is reversed after the first oxidation peak of EUG, 4-allyl-1,2-diquinone is reduced to 4-allyl-1,2-hydroquinone involving two electrons and two protons, which is oxidized to the original quinone during the second potential sweep in a quasi-reversible process (Figure 4a,b).

On the other hand, if the direction of the potential sweep is reversed at 0.55 V during the first oxidation cycle of EUG, the IIa/IIc redox couple is not observed. This clearly shows that the process leading to peak Ia is responsible for the appearance of IIa/IIc redox couple at lower potentials than peak Ia.

These results are in good agreement with a previous report where the formation of both products was postulated using computational methods and proved by Mass spectroscopy experiments [26]. As a humble reflection about this point, mass spectrometry shows all thermodynamically feasible products in a huge time window; being contrary to the short times of the CV experiments where the kinetics govern the process. Said that previous reports are in agreement with the fact that the electrochemical behavior observed for the oxidation product of EUG is consistent with the well-studied *o*-diquinone (in this case, 4-allyl-1,2-diquinone, DQ) [32,33].

### 2.5. Kinetic Parameters of the EUG Oxidation Product (DQ)

**Figure 5:**
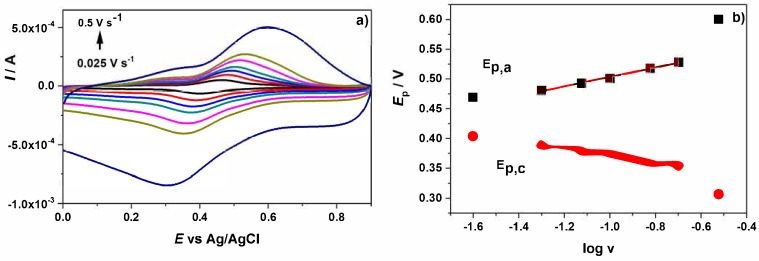
(**a**) Cyclic voltammograms recorded for EUGOP (DQ) at different v (0.025, 0.05, 0.075, 0.1, 0.15, 0.2 and 0.5 V s^−1^) at GCE/ePRGO in pH 2.0 PBS. (**b**) Plots of E_p,a_ and E_p,c_ as a function of log v. C_EUG_ = 1 × 10^−3^ M in solution for the accumulation.

After performing the accumulation of EUG at GCE/ePRGO the electrode was rinsed with the buffer solution and transferred to an electrochemical cell containing only the blank solution (pH 2.0 PBS). Under these conditions, cyclic voltammograms were recorded at different v, in the range from 0.025 to 0.500 V s^−1^. Figure 5a shows cyclic voltammograms obtained for the EUGOP (DQ). Figure 5b shows the dependence of E_p,a_ and E_p,c_ as a function of log v for the EUGOP (DQ).

According to a methodology proposed by Laviron [34] for quasi-reversible couples, where both components are strongly adsorbed at the electrode surface, it is possible to determine the kinetic parameters such as the overall heterogeneous rate constant ($k_{s}$) and the anodic transfer coefficient (1 − α). (1 − α) was calculated from the slope of the linear portion of the E_p,a_ vs. log v plot through Equation (1):

(1) $$\frac{dE_{p,a}}{\mathrm{dlog}\;v}=\frac{2.303\;R\;T}{\left(1-\alpha \right)n\;F}\;\;$$

where R = 8.314 J mol^−1^ K^−1^, T = 298 K, F = 96484.6 C and n = 2. On the other hand, $k_{s}$ was calculated from the following equation (Equation (2)):

(2) $$\log k_{s}=\alpha \log\left(1-\alpha \right)+\left(1-\alpha \right)\log\alpha -\log\left(RT/nFv\right)-\alpha nF\Delta E_{p}\left(1-\alpha \right)/2.303\;RT$$

$k_{s}$ was calculated in the v range from 0.050 to 0.2 V s^−1^. Values of $k_{s}$ and (1 − α) were (2.6 ± 0.2) s^−1^ and 0.41, respectively.

### 2.6. DFT Calculations and CV Simulations

**Scheme 3:**
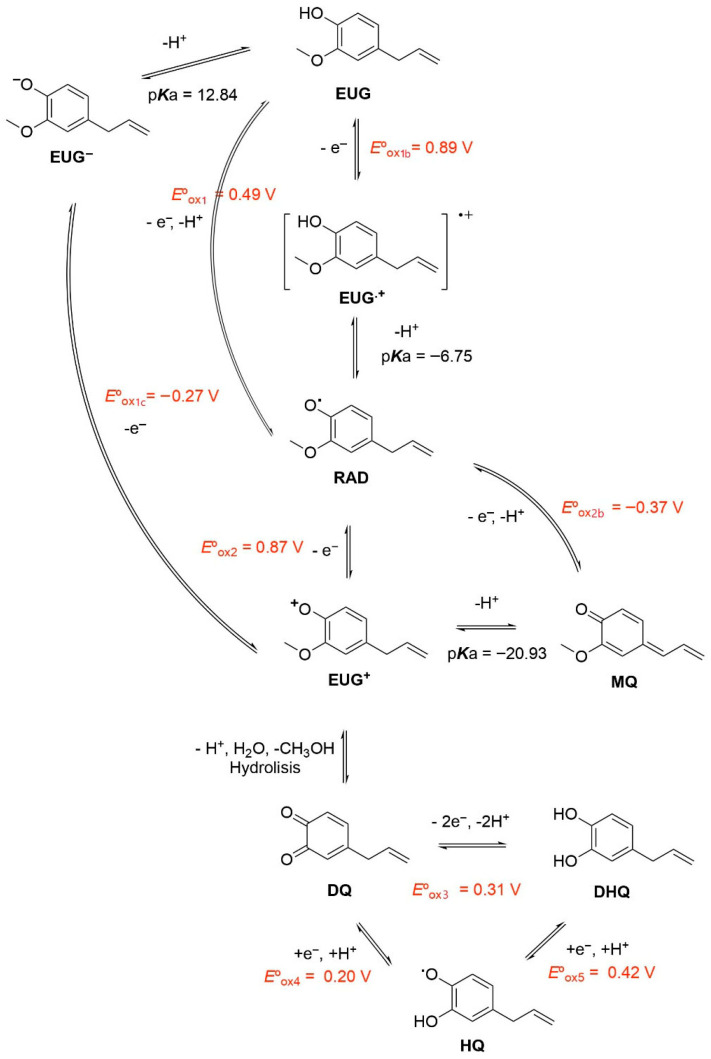
EUG proposed pathways for oxidation mechanism.

From Scheme 1 we studied the oxidation mechanism of EUG with DFT from the calculations of oxidation potentials and pKa’s. The possible pathways are shown in Scheme 3. For the sake of simplicity of notation, it was necessary to label the compounds with a characteristic symbol. We proposed the following notations: Eugenol (EUG), cation radical (EUG^+^), Anion (EUG^−^), radical (RAD), cation (CAT), methylenequinone (MQ), 4-allyl-1,2-diquinone (DQ), 4-allyl-1,2-hydroquinone (DHQ), the intermediate between DQ and DHQ (HQ). Also, the potentials for different redox reactions were labelled with a symbol.

We considered that the oxidation of EUG preferably follows the pathway called A in Scheme 1 (radical formation over the oxygen group), therefore the charge passing through B was considered to be negligible. This is understood from an organic point of view because the EUG is oxidized loosing one electron the radical cation formed is located over the oxygen atom and the aromatic ring and none contribution is observed over the allilyc carbon. By other way, the formation of the anionic form of EUG requires high pHs since the calculated pKa was 12.84 (close to the experientially obtained). When oxidating EUG to form RAD two possible pathways may occur: (1) an electron loss followed by a deprotonation and (2) a concerted proton/electron transfer. The second one would be preferred since presents a lower potential value of 0.49 V, this value is close to the experimental potential peak. When RAD is formed the oxidation of this species may follow two possible paths. We considered that both may occur. By one side, the oxidation of the formation of MQ in a concerted proton/electron transfer reaction shows a very low standard potential, this means that when RAD is formed the electrode potential is well above the standard potential of the redox couple and the reaction immediately occur. By the other side, the formation on CAT is expected since this is the only way to produce the electroactive species DQ. The formation of CAT is favored because it is followed by a hydrolysis reaction, which is strongly promoted by being in an aqueous medium. CAT may also loss a proton to form MQ. Finally, once DQ is formed, can be reduced to DHQ by two possible paths, in one step or in two different steps.

According to previous DFT results, we studied the voltammetric responses of EUG and its derivates with continuum voltammetry simulations. Thus, in order to study the electrochemistry of eugenol in GCE/ePRGO, pH 2.0 PBS, after 20 min of accumulation time, it was assumed that all molecules (EUG and its products) are adsorbed on the surface during the experimental time ($\Gamma_{EUG}$ is the surface concentration of EUG). For the modeling, it was also considered that the diffusive current is negligible compared to the adsorbed one.

**Scheme 4:**
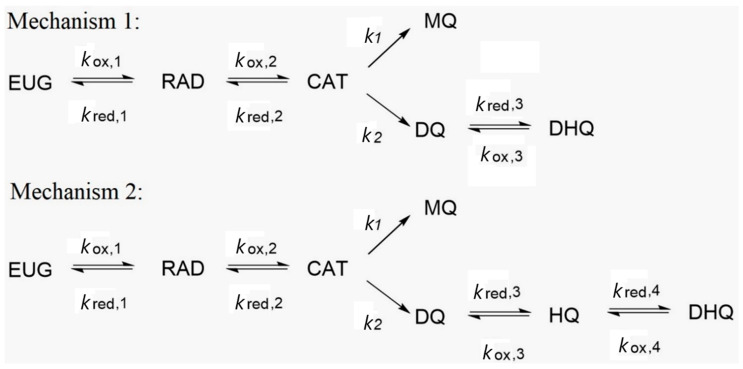
Two possible pathways for the final reaction of DQ.

The considered mechanisms for voltammetry simulations are detailed in Scheme 4. The systems of equations for the reactions are detailed in the following:

**Mechanism 1 in** Scheme 4**:**

(3) $$\frac{d\Gamma_{EUG}}{dt}=-\Gamma_{EUG}k_{ox,1}+\Gamma_{RAD}k_{red,1}\;$$

(4) $$\frac{d\Gamma_{RAD}}{dt}=\Gamma_{EUG}k_{ox,1}-\Gamma_{RAD}k_{red,1}-\Gamma_{RAD}k_{ox,2}+\Gamma_{CAT}k_{red,2}$$

(5) $$\frac{d\Gamma_{CAT}}{dt}=\Gamma_{RAD}k_{ox,2}-\Gamma_{CAT}k_{red,2}-\Gamma_{CAT}k_{1}-\Gamma_{CAT}k_{2}\;$$

(6) $$\frac{d\Gamma_{MQ}}{dt}=\Gamma_{CAT}k_{1}$$

(7) $$\frac{d\Gamma_{DQ}}{dt}=\Gamma_{DHQ}k_{ox,3}-\Gamma_{DQ}k_{red,3}+\Gamma_{CAT}k_{3}$$

(8) $$\frac{d\Gamma_{DQ}}{dt}=\Gamma_{DHQ}k_{ox,3}-\Gamma_{DQ}k_{red,3}+\Gamma_{CAT}k_{3}$$

**Mechanism 2 in** Scheme 4**:**

(9) $$\frac{d\Gamma_{EUG}}{dt}=-\Gamma_{EUG}k_{ox,1}+\Gamma_{RAD}k_{red,1}\;$$

(10) $$\frac{d\Gamma_{RAD}}{dt}=\Gamma_{EUG}k_{ox,1}-\Gamma_{RAD}k_{red,1}-\Gamma_{RAD}k_{ox,2}+\Gamma_{CAT}k_{red,2}$$

(11) $$\frac{d\Gamma_{CAT}}{dt}=\Gamma_{RAD}k_{ox,2}-\Gamma_{CAT}k_{red,2}-\Gamma_{CAT}k_{1}-\Gamma_{CAT}k_{2}\;$$

(12) $$\frac{d\Gamma_{MQ}}{dt}=\Gamma_{CAT}k_{1}$$

(13) $$\frac{d\Gamma_{DQ}}{dt}=\Gamma_{HQ}k_{ox,3}-\Gamma_{DQ}k_{red,3}+\Gamma_{CAT}k_{2}$$

(14) $$\frac{d\Gamma_{HQ}}{dt}=-\Gamma_{HQ}k_{ox,3}+\Gamma_{DQ}k_{red,3}+\Gamma_{DHQ}k_{ox,4}-\Gamma_{HQ}k_{red,4}$$

(15) $$\frac{d\Gamma_{DHQ}}{dt}=-\Gamma_{DHQ}k_{ox,4}+\Gamma_{HQ}k_{red,4}$$

In these equations, $\Gamma_{i}$ represent the surface concentrations of the *i*-th species, $k_{ox,j}\;$ and $k_{red,j}$ are the oxidation and reduction rate constants of the *j*-th reaction and $k_{h}$ denotes the rate constant of the *h*-th chemical reaction.

Being the total current the summation of the partial currents calculated with the BV equation (Equation (26)).

**Mechanism 1:**

(16) $$I_{T}=I_{1}+I_{2}+I_{3}$$

**Mechanism 2:**

(17) $$I_{T}=I_{1}+I_{2}+I_{3}+I_{4}$$

**Figure 6:**
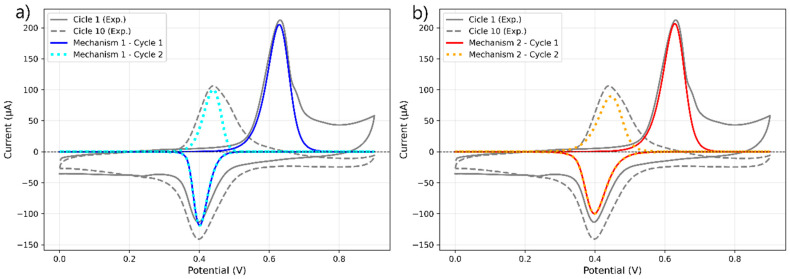
Experimental and numerical simulation of EUG in GCE/ePRGO, pH 2.0 PBS, at 0.1 V s^−1^. For cycles indicated in the figure. (**a**) Mechanism M1, (**b**) Mechanism M2, detailed in Scheme 3.

Figure 6 presents the simulated voltammetric responses obtained using **Mechanism 1** (panel a) and **Mechanism 2** (panel b). The kinetic and thermodynamic parameters employed for each mechanism are summarized in Table 1 and Table 2, respectively. For the DQ-related reactions, the parameters described in Section 2.5 were used. The reaction pathway from RAD to MQ was excluded from the model because its inclusion led to voltammetric responses that were inconsistent with the experimental data. A brief discussion of the mechanism including this pathway is provided in the Supporting Information (Section S2). Also, an error analysis of the simulated voltammograms is shown in Section S2.

As observed in both panels, the simulated anodic peak I corresponding to EUG oxidation accurately reproduces the experimental response. However, differences arise in the cathodic region. In particular, for the reduction of 4-allyl-1,2-diquinone (DQ), Mechanism 2 reproduces the shape and position of peak IIc more satisfactorily than Mechanism 1. These results suggest that DQ reduction proceeds through two consecutive one-electron steps rather than a single two-electron step. Such behavior has been previously reported for diquinones in general [35,36].

The simulations further indicate that all adsorbed EUG is consumed during the formation of peak I, yielding MQ and DQ as oxidation products. The DQ generated during the partial oxidation of EUG subsequently participates in the reverse scan. Since DQ formation from CAT is modeled as an irreversible process, the same amount of charge contributes in subsequent cycles to the formation of peaks IIa/IIc. This is consistent with the experimental observation that peak I, corresponding to EUG oxidation, is absent in cycles following the first one.

The previous description shows that what happens in the proposed model for peaks IIa/IIc, is slightly different from the experiment, where the current of peaks IIa/IIc barely increases with the cycles. This means that adsorption of species may be present, but coupled with a source of EUG. For example, by a solution contribution or adsorption-desorption dynamics that are not considered in our model

## 3. Materials and Methods

### 3.1. Reagents

EUG was purchased from Sigma–Aldrich. Ethanol, NaH_2_PO_4_, Na_2_HPO_4_, HCl, and NaOH were Merck p.a. Ultrapure water (ρ = 18 MΩ cm) was obtained from a Millipore-Milli Q system. The EUG stock solution was 0.64 M in ethanol. It was protected from light and kept in the refrigerator. The corresponding working solutions were prepared daily by adding different aliquots of the stock solution to the corresponding reaction media. All reagents were used as received.

PBS solutions of different pH values were prepared using 0.1 M K_2_PO_4_H/KPO_4_H_2_ adjusting the pH by adding different volumes of 1 M HCl or 1 M NaOH solutions as corresponding.

### 3.2. Apparatus and Experimental Measurements

Cyclic voltammetry (CV) experiments were carried out with an AutoLab PGSTAT 101 potentiostat, controlled by NOVA 1.9 electrochemical software from Eco-Chemie, Utrecht, The Netherlands. The scan rate (v) was varied from 0.025 to 0.5 V s^−1^. Electrochemical impedance spectroscopy (EIS) was carried out with a PalmSens4 Potentiostat.

Electrochemical measurements were performed in a 5 mL Pyrex cell. Working electrodes were either a GCE (CH Instruments, 3 mm diameter), GCE/GO, or a GCE/ePRGO. A platinum wire and Ag/AgCl (3 M NaCl) (BAS, RE-5B) were used as counter and reference electrodes, respectively.

The working temperature was 25.0 ± 0.1 °C. Theoretical calculations were made using the Density Functional Theory method for the optimization of structures of each one of the possible oxidation products proposed.

EIS measurements were performed in solutions containing equal concentrations (1 × 10^−3^ M) of reduced and oxidized forms of the [Fe(CN)_6_]^4−/3−^ redox couple, with 0.1 M KCl as the supporting electrolyte. An amplitude sine wave perturbation of 5 mV was applied to electrodes, whereas the dc potential was set at the formal potential of the redox couple, i.e., E_f_^o^ = 0.250 V vs. Ag/AgCl. The ac frequency was varied from 0.5 Hz to 10 kHz. Both, the in-phase (Z′) and out-phase (Z″) impedance components were extracted from experimental data.

SEM measurements (Carl Zeiss Sigma with Zeiss-MMSTEM detector) were used to characterize the different stages during the modification of the electrode surface.

### 3.3. Computational Details

The methodology used in the present article is adapted and modified from prior studies [37,38,39]. This computational method allows performing simulations of CV to contrast the theoretical predictions with the voltammetric experiments from predicted mechamisms with DFT calculations.

The methodology used in the present article is adapted and modified from prior works [37,38,39,40]. The calculations were performed by the Gaussian 09 Rev. E01 series of programs using the global hybrid functional B3LyP and 6–31+G(d) as a basis set, and the SMD implicit solvation model. This functional has been evaluated previously and was concluded to have a consistent and uniform response both in reductions and oxidations in aqueous systems, having an adequate balance between accuracy and computational effort [41]. In addition, geometries were fully optimized in the water solution. Harmonic frequencies were calculated to confirm the structures were minima on the potential energy surface and to obtain thermal and entropic contributions to the free energies.

The p*K*a calculation was based on the proton dissociation reaction shown in Equation (18) [42,43]. The p*K*a of molecule HA was calculated according to Equation (19), where G*_aq_,A^−^ and G*_aq_,HA are the standard free energy of deprotonated and protonated species, respectively calculated directly in aqueous solution at 298.15 K.

(18) $${HA}_{aq}\overset{{∆G}_{aq}^{*}}{\to }\;A_{aq}^{-}+H_{aq}^{+}$$

(19) $$pKa=\frac{{∆G}_{aq}^{*}}{2.303RT}=\frac{G_{aq,A-}^{*}+G_{aq,H+}^{*}-G_{aq,HA}^{*}}{2.303RT}$$

The Gibbs free energy of a proton used was −270.29 kcal/mol [42,43,44]. Δ*G*1atm⟶1*M* = *RT* (24.46) = 1.89 kcal/mol is a correction term for the change in a standard state of 1 atm to 1 mol/L. The symbols * and o denote the standard state of 1 mol/L and 1 atm, respectively.

For a reduction reaction,

(20) $$A_{aq}+ne^{-}\overset{{∆G}_{red(aq)}^{*}}{\to }A_{aq}^{n-}$$

The standard reduction potential is

(21) $$E_{red(aq)}^{{}^{\circ}}=-\frac{{∆G}_{red\left(aq\right)}^{*}}{nF}-SHE$$

where ∆G*_red(*aq*)_ is the Gibbs free energy of the reduction in standard conditions, n is the number of electrons transferred in the reduction process, F is Faraday’s constant (23.06 kcal (mol V^−1^)), and SHE is the absolute potential of the standard hydrogen electrode (4.281 V) [44,45]. 0.2 V was used for the correction for SHE to Ag/AgCl electrode. The Gibbs free energy for reduction is

(22) $${∆G}_{red(aq)}^{*}=G_{aq}\left(A^{n-}\right)-G_{aq}\left(A\right)-{nG}_{g}^{{}^{\circ}}(e^{-})$$

where $G_{g}^{{}^{\circ}}(e^{-})$ is the gas-phase free energy of one electron. At 298 K, the gas phase Gibbs energy of an electron is $G_{g}^{{}^{\circ}}\left(e^{-}\right)=0.867\;kcal/mol$ and is obtained from the literature values of $H_{g}^{{}^{\circ}}\left(e^{-}\right)=0.752\;kcal/mol$ and $S_{g}^{{}^{\circ}}\left(e^{-}\right)=5.434\;kcal/mol\;K$ [46].

All voltammetry simulations were performed using an ad hoc code written in Python 3.0 programming language. The concentration of adsorbed species Γ on the electrode surface, changes with time according to the general equation:

(23) $$\frac{d\Gamma }{dt}=-\sum_{j}\Gamma_{R}k_{ox,j}+\;\sum_{j}\Gamma_{O}k_{red,j}\pm S_{i}$$

In this equation, *j* is a label for redox reactions, the electrochemical rate constants ($k_{ox,j}\;\mathrm{and}\;k_{red,j}$) were calculated with the BV approach:

(24) $$k_{ox,j}=k_{s,j}e^{\left(1-\alpha_{j})f(E_{app}-{E_{j}}^{0}\right)}$$

(25) $$k_{red,j}=k_{s,j}e^{-\alpha_{j}f(E_{app}-{E_{j}}^{0})}$$

with $f=n_{j}F/(RT)$ and $k_{s,j}$ being the heterogeneous rate constant, $\alpha_{j}$ the transfer coefficient, $E_{app}$ the applied potential and ${E_{j}}^{0}$ the standard potential.

The faradaic current for each redox couple is then calculated with:

(26) $$I_{j}=n_{j}FS(\Gamma_{R}k_{ox,i}-\Gamma_{O}k_{red,i})\;$$

Being the total current the summation of the partial currents as following

(27) $$I_{T}=\sum I_{j}$$

Then $S_{i}$ is a source of species from non-redox first-order chemical reaction with rate constant $k_{i}$ and species concentration Γ is given by the following equation:

(28) $$S_{i}=\Gamma k_{i}$$

The derivates were solved with the two-point finite differences method, considering a sufficient large number of time points to ensure minimizing the numerical error of the approximation.

### 3.4. Procedure for Preparation of Electrodes

*Pretreatment of GCE*: the electrodes were polished with alumina slurries of 0.30 and 0.05 µm for 1 min each one, and sonicated in water for 30 s. The GCE electrochemical area was determined by chronoamperometric measurements using Cottrell plots [47] in 1 × 10^−3^ M K_4_[Fe(CN)_6_] + 0.5 M KNO_3_, using a diffusion coefficient of 7.6 × 10^−6^ cm^2^ s^−1^ for K_4_[Fe(CN)_6_] [48]. An average value of A = (0.089 ± 0.004) cm^2^ was determined from measurements carried out with the same electrode in triplicate.

*Preparation of GCE/GO*: the polished GCE was modified with the GO dispersion by dropping an aliquot of 10 µL of the dispersion on the top of the electrode and allowing drying for 30 min at 37 °C. The GO dispersion was synthesized following the same procedure as it was previously described [49].

*Preparation of GCE/ePRGO*: the modified electrode was generated by cycling the potential (30 cycles) between −0.200 V and −1.7 V at 0.052 V s^−1^ on GCE/GO in 0.02 M pH 7.0 PBS. The optimal conditions to generate the GCE/ePRGO were chosen using an experimental design as it was previously described [39]. On the other hand, an electro-active area of 1.4 cm^2^ was obtained for the GCE/ePRGO.

## 4. Conclusions

The electro-oxidation of eugenol was studied at a glassy carbon modified with electrochemically partially reduced graphene oxide in phosphate buffer solutions of different pH by using cyclic voltammetry. The cyclic voltammograms were much better defined at glassy carbon electrodes modified with electrochemically partially reduced graphene oxide, showing that the electrode process is mainly controlled by adsorption. The main oxidation product gives rise to a quasi-reversible redox couple, which was assigned to the 4-allyl-1,2-diquinone/4-allyl-1,2-dihydroquinone redox couple. From cyclic voltammograms recorded in blank solutions for the eugenol oxidation product, the kinetic parameters such as the overall heterogeneous rate constant and the anodic transfer coefficient were determined. The oxidation mechanism was then analyzed with DFT calculations and modelled with continuum simulations of adsorbed species to contrast the proposed pathways with the experimental voltammograms.

The proposed mechanisms describe a multi-step redox sequence in which Eugenol is reversibly oxidized to the radical species and then to the cationic one via two consecutive electron transfers. From the cationic one, the system branches into two competing chemical pathways leading to a methilquinone and a 4-allyl-1,2-diquinone. The fundamental difference between the two proposed mechanisms lies in the electrochemical reaction of 4-allyl-1,2-diquinone: in one of the pathways this molecule is directly reduced to 4-allyl-1,2-hydroquinone in a single reversible electrochemical step (Mechanism 1), while in the other the reduction occurs in a stepwise fashion, first passing through an hydroquinone intermediate before reaching4-allyl-1,2-hydroquinone (Mechanism 2). This distinction implies different kinetics and electrochemical signatures, associated with the separation of the electron transfer processes. According to our simulation results, the current response predicted by Mechanism 2 closely matches the experimental response. As observed, the current obtained from Mechanism 1 exhibits a Peak II with a narrower half-peak width compared to Mechanism 2, strongly suggesting that the reduction of diquinone proceeds through two redox processes rather than a single one.

The coincidence between the theoretical model and the experiments show that the proposed mechanisms explain the electrochemistry of adsorbed eugenol and the oxidation products involved on partially reduced graphene oxide.

## Figures and Tables

**Table 1:** Mechanism 1 parameters used for calculations.

Parameter	Value
Sweep rate	0.1 V/s
Electrode surface	0.105 cm^2^
$\Gamma_{EUG}$	8.3 × 10^−9^ mol/cm^2^
${E_{1}}^{0}$,${E_{2}}^{0}$, ${E_{3}}^{0}$	0.61 V, 0.625 V, 0.42 V
$k_{s,1}$, $k_{s,2}$,$\;k_{s,3}$	10 s^−1^, 10 s^−1^, 2.8 s^−1^
$\alpha_{1}$, $\alpha_{2}$, $\alpha_{3}$	0.5, 0.5, 0.59
$n_{1}$, $n_{2},n_{3}$	1, 1, 2
$k_{1}$, $k_{2}$,	1.0 s^−1^, 0.6 s^−1^

**Table 2:** Mechanism 2 parameters used for calculations.

Parameter	Value
Sweep rate	0.1 V/s
Electrode surface	0.105 cm^2^
$\Gamma_{EUG}$	8.3 × 10^−9^ mol/cm^2^
${E_{1}}^{0}$,${E_{2}}^{0}$, ${E_{3}}^{0}$, ${E_{4}}^{0}$	0.61 V, 0.625 V, 0.42 V, 0.425 V
$k_{s,1}$, $k_{s,2}$,$\;k_{s,3},\;k_{s,4}$	10 s^−1^, 10 s^−1^, 5 s^−1^, 5 s^−1^
$\alpha_{1}$, $\alpha_{2}$, $\alpha_{4},\;\alpha_{4}$	0.5, 0.5, 0.59, 0.59
$n_{1}$, $n_{2}$, $n_{3},n_{4}$	1, 1, 1, 1
$k_{1}$, $k_{2}$,	1.0 s^−1^, 0.9 s^−1^

## Data Availability

The original contributions presented in this study are included in the article. Further inquiries can be directed to the corresponding authors.

## Supplementary Materials

The following supporting information can be downloaded at: https://www.mdpi.com/article/10.3390/ijms27052461/s1.
