# Supplementary material for: Understanding the Oxidation Electrochemistry of Adsorbed Eugenol on a Glassy Carbon Electrode Modified with Electrochemically Partially Reduced Graphene Oxide: A Theoretical and Experimental Approach
Source: Int J Mol Sci. 2026 Mar 7;27(5):2461. doi: 10.3390/ijms27052461 (PMC12985879; doi:10.3390/ijms27052461)
Supplement: Supplementary file 1 [file ijms-27-02461-s001.zip › ijms-4181813-supplementary.pdf]

SUPPLEMENTARY MATERIAL

Understanding the Oxidation Electrochemistry of Adsorbed Eugenol on a Glassy Carbon Electrode Modified with Electrochemically Partially Reduced Graphene Oxide: A Theoretical and Experimental Approach

Gastón Darío Pierini [1,*], Edgardo Maximiliano Gavilán-Arriazu [2], Sergio Antonio Rodriguez [3], Sebastián Noel Robledo [4], Héctor Fernández [1] and Adrian Marcelo Granero [1,*]

[1] Grupo de Electroanalítica (GEANA), Departamento de Química, Facultad de Ciencias Exactas, Físico-Químicas y Naturales, Instituto para el Desarrollo Agroindustrial y de la Salud (IDAS), Universidad Nacional de Río Cuarto, Río Cuarto 5800, Argentina

[2] Instituto de Bionanotecnología del NOA (INBIONATEC), Universidad Nacional de Santiago del Estero (UNSE), Santiago del Estero 4200, Argentina; maxigavilan@gmail.com

[3] Instituto de Ciencias Químicas, Facultad de Agronomía y Agroindustrias (FAyA), Universidad Nacional de Santiago del Estero (UNSE), CONICET, Av. Belgrano Sur 1912, Santiago del Estero 4200, Argentina

[4] Departamento de Tecnología Química, Facultad de Ingeniería, Instituto para el Desarrollo Agroindustrial y de la Salud (IDAS), Universidad Nacional de Río Cuarto, Río Cuarto 5800, Argentina

* Correspondence: gpierini@exa.unrc.edu.ar (G.D.P.); agranero@exa.unrc.edu.ar (A.M.G.)

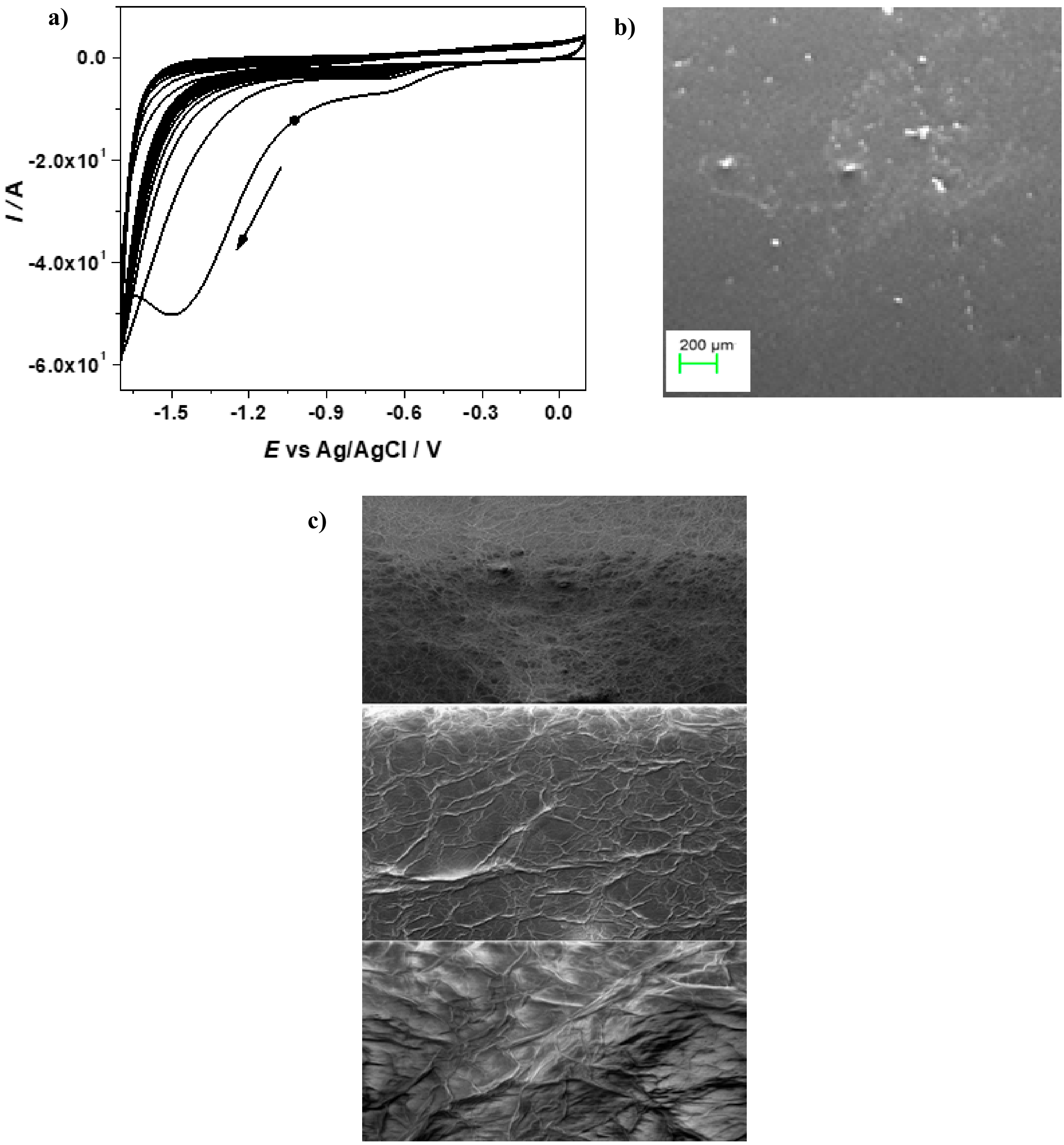

Figure S1. a) Cyclic voltammograms recorded in 0.2 mol L^{-1} phosphate buffer solution (pH 7.0) using GCE/OG. v = 0.052 V s^{-1}. SEM images of b) GCE, and c) GCE/ePRGO at different magnifications.

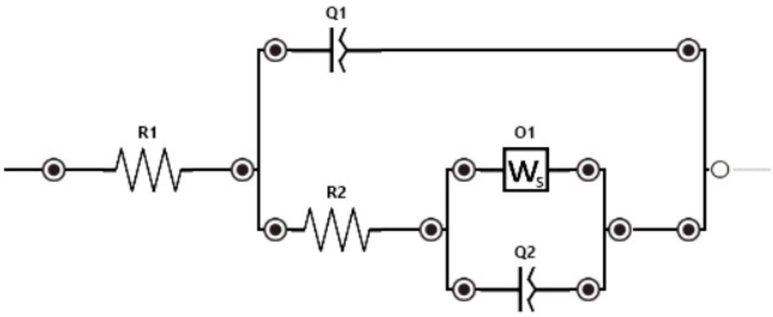

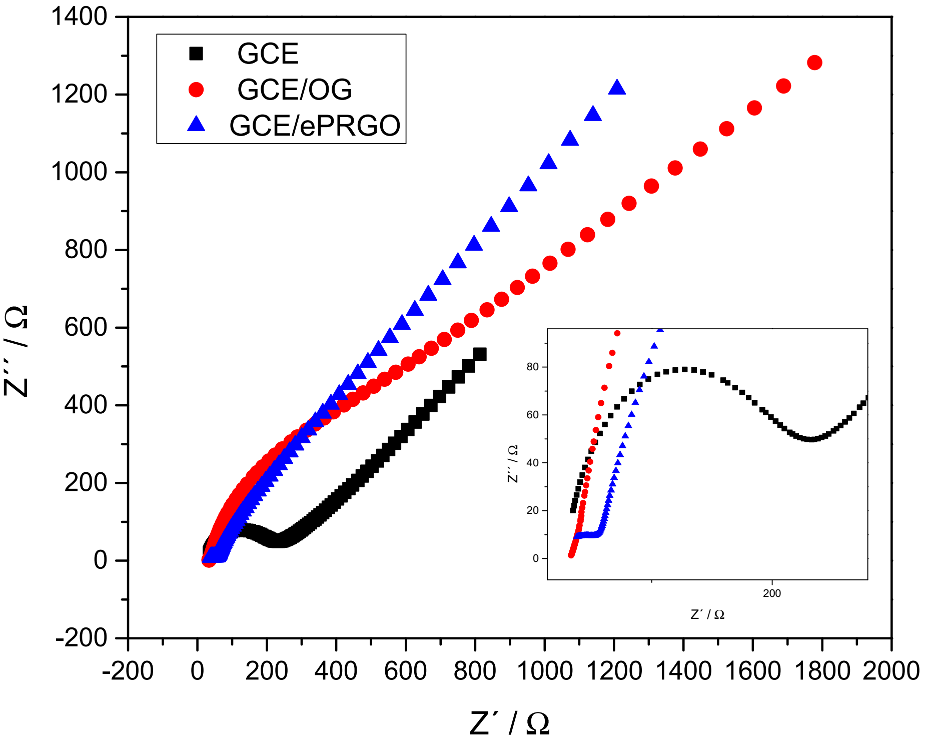

Figure S2. Nyquist plots for GCE (black points), GCE/ePRGO (blue points) and GCE/OG (red points), obtained in the presence of 1.0×10^{-3} M of reduced and oxidized forms of $[Fe(CN)_6]^{4-/3-}$ redox couple + 0.1 M KCl. Inset show amplification of Nyquist plots.

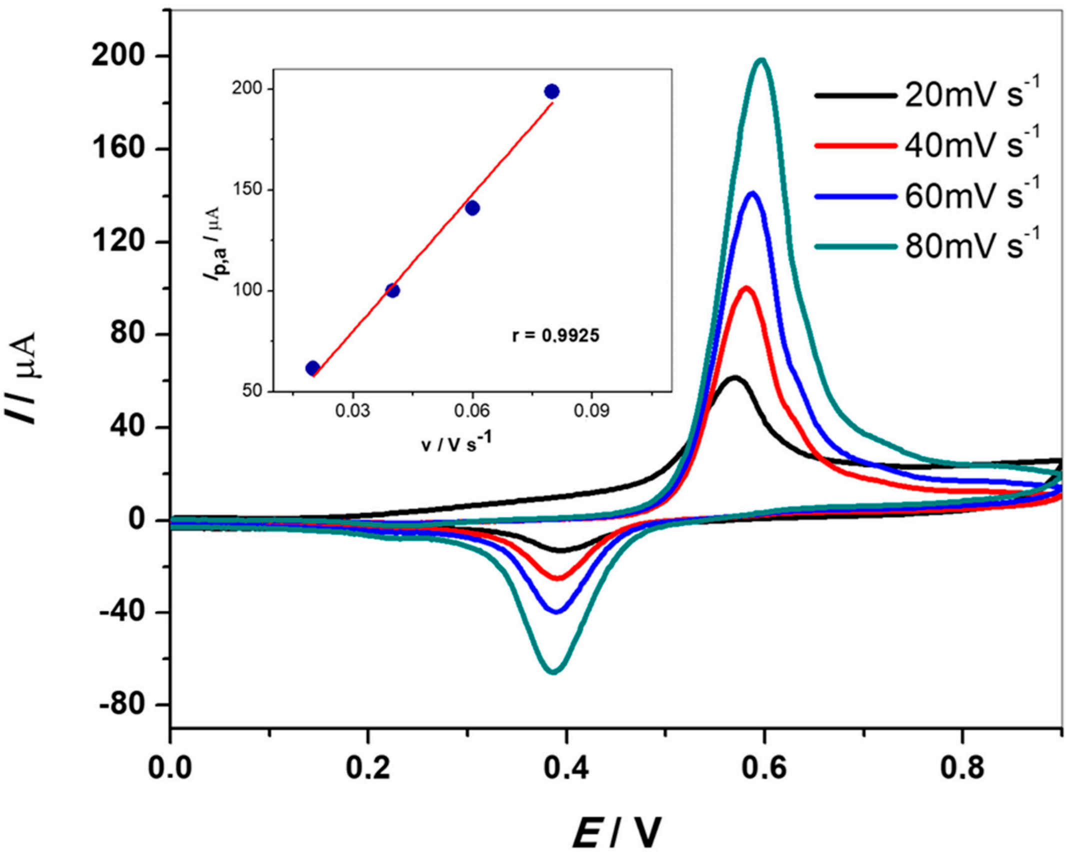

Figure S3. Cyclic voltammograms of 5 x 10^{-4} M EUG in 0.2 M pH 2.0 PBS at GCE/ePRGO. At different sweep rates. The $I_{p,a}$ vs *v* plot is shown in the inset.

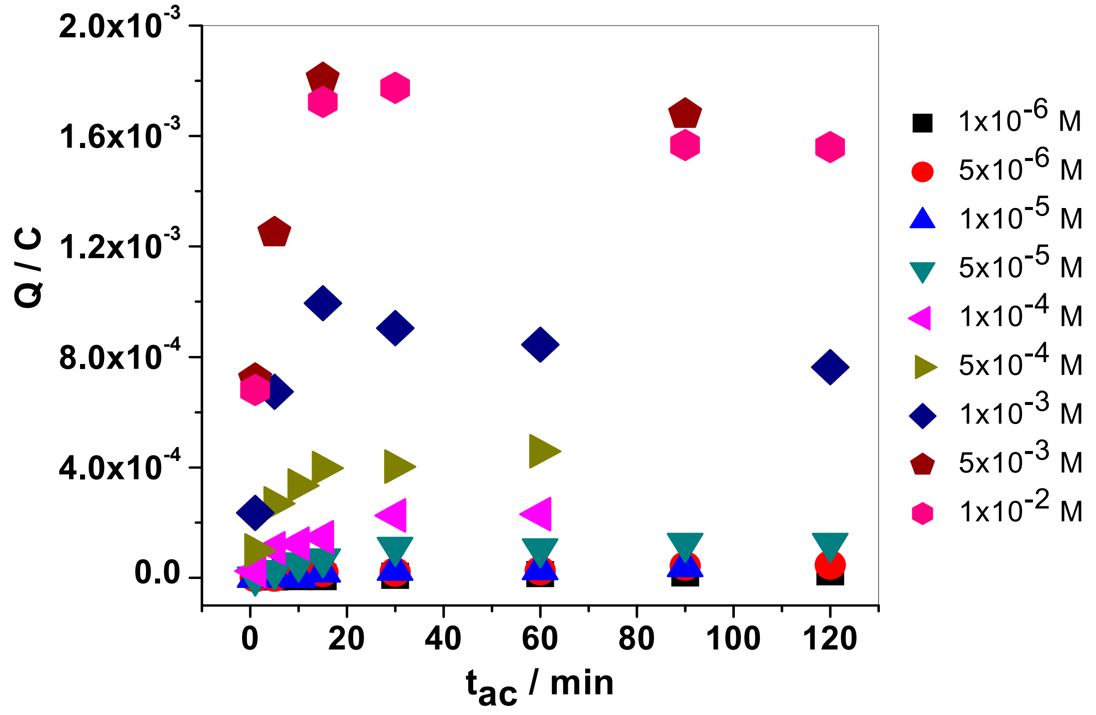

Figure S4. Charge transferred for different bulk solution concentration and accumulation times (t_{ac}).

S1. Mechanism with the pathway from RAD to MQ

In this section we discuss the inclusion of the pathway from the radical (RAD) to the methylquinone (MQ) species, labelled with E^0_{2b} in Scheme 4. The complete mechanism (Mechanism 3) is detailed in Scheme S1. The results for simulations performed with the parameters shown in Table S3 for different $k_{s,2b}$. show that, as commented in the manuscript, the voltammetric response does not coincides with the experimental voltammetry. As observed, in all cases the reaction of the quinone is not present, except for the lowest rate constant used where a very small pair of peak appears even when we overestimate the rate of cation production compared to previous mechanisms (k_2=30 s^{-1}). Another important fact is that a reductive peak due to the formation of MQ appears at negative potentials. This peak is not observed in experiments [16].

Scheme S1. Mechanism 3 for EUG oxidation mechanism.

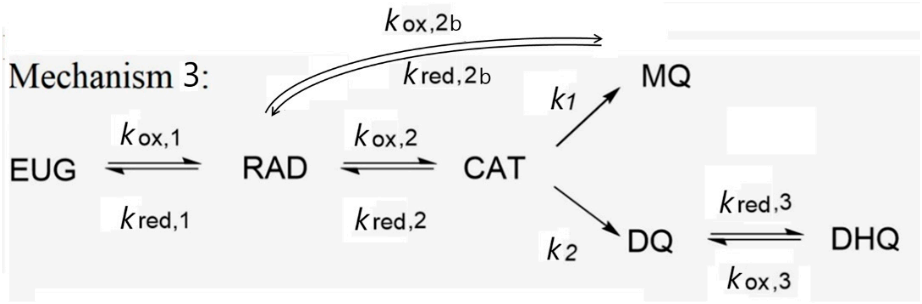

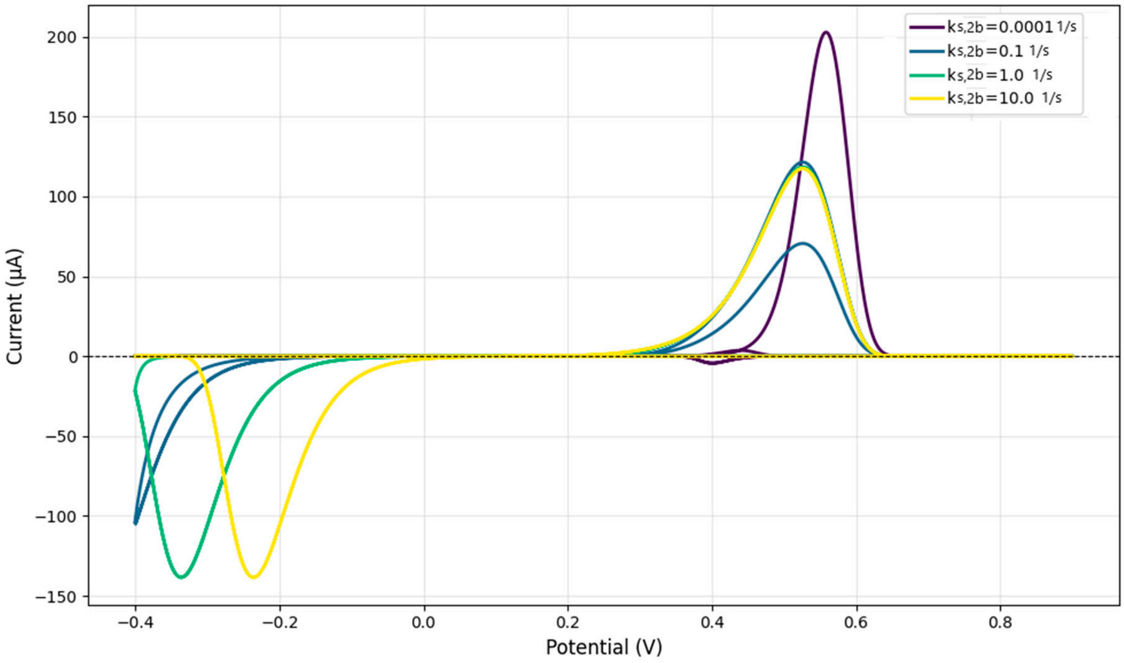

Figure S5. Voltammetric simulation of Mechanism 3 presented in Scheme S1using different values of $k_{s,2b}$.

Table S1. Scheme S1 parameters used for calculations.

**Parameter**	**Value**
Sweep rate	0.1 V/s
Electrode surface	0.105 cm^2
Γ_{EUG}	8.3x10^{-9} mol/cm^2
$E_1{}^0$,$E_2{}^0$, $E_{2b}{}^0$, $E_3{}^0$	0.61 V, 0.625 V, -0.31V 0.42 V
$k_{s,1}$, $k_{s,2}$, $k_{s,3}$	10 s^{-1}, 10 s^{-1}, 2.8 s^{-1}
α_1, α_2, α_{2b} α_3	0.5, 0.5, 0.5, 0.59
n_1, n_2, n_{2b}, n_3	1, 1, 1, 2
k_1, k_2,	1.0 s^{-1}, 30 s^{-1}

S2. Simulations error analysis

Figure S6 shows the residual analysis for peak I in a potential window from 0.5 V to 0.65 V. This analysis was not performed for peak II because this voltammetric wave naturally emerges using the calculated experimental parameters and setting the rate constant values for chemical processes. The residuals are represented as relative and absolute values, according to Equation S1.

$$residual = \left|\frac{I_{th}-I_{exp}}{I_{exp}}\right| x100 \qquad \text{(S1)}$$

where I_{th} is the theoretical current and I_{exp} is the experimental current.

The experimental and simulated peak I in cycle 1 is shown above the residual figure for reference. As expected, for small experimental currents the residuals present large relative errors. The relative error gradually decreases until a minimum value at the maximum current (small than 5%). The relative error increases with the potential after this minimum. The differences after 0.65 V are larger since a small shoulder is observed in the experimental current, shoulder that it is not reproduced by the model.

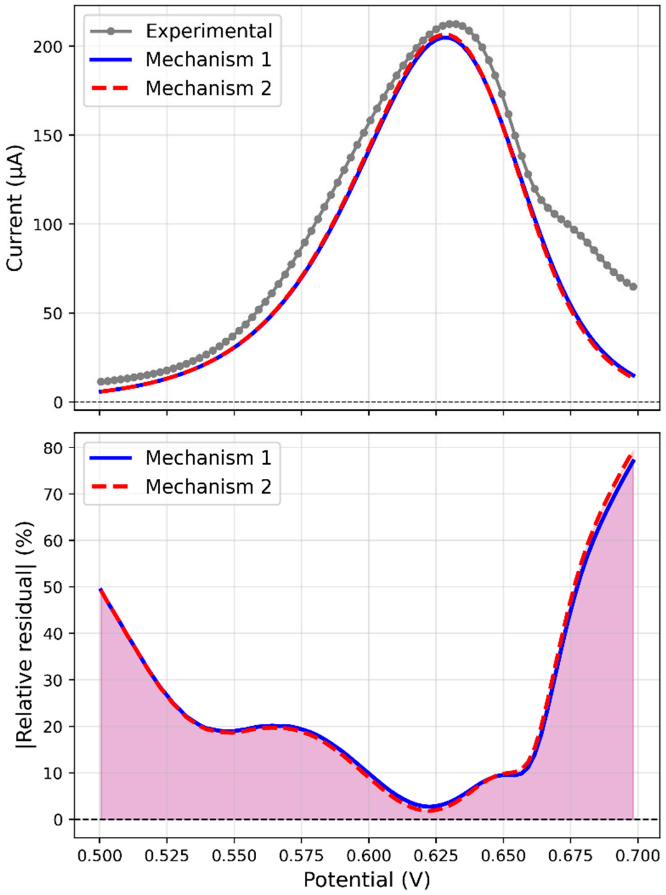

Figure S6. Residual analysis for the oxidative peak I.

Given the behavior of the residuals, the Root Mean Square Error (RMSE) is sensitive to the data considered. This is why, it is most convenient to exclude the current that deviates from

the model and the RMSE was calculated for a potential window of 0.5 V to 0.65 V. The results for this potential window, using 62 experimental data points are:

Mechanism 1: RMSE = 11.4900 μA

Mechanism 2: RMSE = 10.8884 μA

References

[16] Sağlam, Ö., Dilgin, D. G., Ertek, B., & Dilgin, Y. (2016). Differential pulse voltammetric determination of eugenol at a pencil graphite electrode. *Materials Science and Engineering: C*, *60*, 156-162.
